# A Cross-Sectional Survey of Factors Affecting Motivation to Undergo Vasectomy: Does the Dobbs v. Jackson Women’s Health Organization Decision Matter?

**DOI:** 10.7759/cureus.108486

**Published:** 2026-05-08

**Authors:** Rebecca Spinaris, Robert T Strait, Jacqueline Fannin, Matthew DeSanto, Nathan Hale

**Affiliations:** 1 Urology, ECU Health, Greenville, USA; 2 Urology, West Virginia School of Osteopathic Medicine, Lewisburg, USA; 3 Urology, Charleston Area Medical Center, Charleston, USA

**Keywords:** attitudes and practices, health services accessibility, medical abortion, survey research, vasectomy

## Abstract

Purpose: Recent changes in the reproductive healthcare environment in the United States have raised questions about how external factors influence decisions regarding permanent contraception. We explored patient motivations underlying the desire for elective sterilization among patients undergoing vasectomy post the *Dobbs v. Jackson Women’s Health Organization* decision.

Methods: A single-institution retrospective cohort with a prospective cross-sectional survey of patients who underwent vasectomy from July 1, 2022, through January 31, 2023, was conducted. Eligible men were contacted for a post-vasectomy telephone survey to capture patient motivations and experiences regarding their decision to undergo vasectomy.

Results: From surveys of 60 male participants, we identified several factors related to the *Dobbs* decision that were associated with the participants’ decision to undergo a vasectomy. Participants with no children comprised 20% of the sample and reported concerns with accessing abortion care and the recent reversal of *Roe v. Wade*, which was associated with vasectomy choice (p<0.05). Participants who were dating and cohabiting were more likely to be associated with reports of these concerns as influences on their decision (p<0.05).

Conclusions: Access to abortion care and recent Supreme Court decisions appear to be important influences on participants’ decision-making prior to vasectomy, particularly for participants without children. A better understanding of the motivations behind the decision to undergo a vasectomy allows for improved patient counseling regarding the intended permanence of the procedure and improved understanding of the changing reproductive healthcare landscape.

## Introduction

A vasectomy is a surgical procedure that has been performed in the United States for more than a century and is commonly known as a definitive solution for male sterilization [1]. Initially rooted in eugenic practices, its adoption broadened significantly for elective sterilization in the aftermath of World War II. The procedure's simplicity, affordability, and high success rate positioned vasectomy as a more popular choice over tubal ligation for couples desiring a permanent birth control option during that period [2]. This long-standing procedure has been recently brought to the forefront, not solely for its biological implications, but due to sociopolitical factors that have greatly altered the reproductive landscape in America.

June 2022 marked a turning point in the history of reproductive rights in the United States. In the landmark case *Dobbs v. Jackson Women’s Health Organization*, the United States Supreme Court reversed the 1973 *Roe v. Wade* decision that had protected the right to an abortion under the 14th Amendment's implicit right to privacy [3,4]. The Dobbs decision substantially changed the reproductive healthcare landscape, leading to rapid policy changes across states, legal challenges, and increased uncertainty surrounding the provision of reproductive care.

Preliminary investigations of vasectomy interest primarily utilized indirect indicators such as Google search-term analysis. This revealed a striking 121% surge in searches for the term "vasectomy" in 49 of the 50 states after a draft Supreme Court opinion suggesting that *Roe v. Wade* would be overturned was leaked in May 2022 [5]. Additionally, analysis has been conducted on Wikipedia following the final *Dobbs* decision. “Vasectomy” page views increased by 183%, as well as other related searches such as “Intrauterine Device” (80% increase), “emergency contraception” (224% increase), and “tubal ligation” (92% increase) [6]. Several studies have reported increases in vasectomy incidence since *Roe v. Wade* reversal, ranging from 20% to 22.4% [7-10]. While limited, there are also a few studies since the overturning of *Roe v. Wade* that have investigated specific factors related to the increase in vasectomy interest. A multi-institutional analysis of vasectomy before and after *Dobbs* noted that after the *Dobbs* decision, men having vasectomy consults and subsequent vasectomies were more likely to be younger, and married men undergoing vasectomies were more likely to be childless [11].

At the Charleston Area Medical Center in Charleston, West Virginia, United States, the number of vasectomies performed markedly increased in the immediate aftermath of the *Dobbs* decision. A review of our billing records revealed that 46 vasectomy procedures were billed through an office or operative procedure area from October 1, 2021, through April 30, 2022. This increased to 382 vasectomy procedures from July 1, 2022, through January 31, 2023 [Unpublished data via medical records]. The primary aim of this study is to evaluate patient motivations for choosing vasectomy after the Supreme Court decision in the *Dobbs v. Jackson Women’s Health Organization* case in June 2022. Robust studies driven by direct patient data are scarce to date. We aimed to fill this gap and better understand patient-driven decision-making in this new era of reproductive healthcare. Thus, a single-institution, prospective questionnaire exploration of the motivations underlying elective male sterilization was conducted to provide a deeper understanding of the interplay between sociopolitical shifts and individual medical decisions.

## Materials and methods

Study design

A comprehensive methodological approach was used in this study, which employed a prospective patient questionnaire designed to provide an in-depth understanding of motivations behind a recent surge in the incidence of vasectomy, observed anecdotally by participants retrospectively identified. The study was approved by the Charleston Area Medical Center’s Institutional Review Board (study number: 23-939).

Study population

The inclusion criteria for this study were patients who underwent vasectomy between July 1, 2022, and January 31, 2023, at the Charleston Area Medical Center. Exclusion criteria entail any patient under the age of 18. By restricting our study to this period, we limited our analysis to the immediate aftermath of the legislative changes and concerns caused by the *Dobbs* decision and their impact on individual reproductive health decisions.

Using the study's eligibility criteria, eligible patients were identified by electronic medical chart review. Patients were recruited via convenience sampling. Eligible participants were informed of the study's purpose, the voluntary nature of participation, and their right to decline or withdraw. Only patients who gave consent to participate were included as participants in the study.

Study instrument

Data were collected using a structured telephone questionnaire (see Appendices). This questionnaire was constructed to capture data points of participants’ motivation, experiences, and opinions regarding the decision to undergo a vasectomy. By directly engaging with patients through the questionnaire, we aimed to tap into the subjective experiences and personal reasons that might not be reflected in the medical record. This questionnaire was developed de novo for this study, as no validated instrument existed to assess patient motivations for vasectomy in the context of reproductive healthcare access and policy changes. Items were written using neutral, non-leading language and a simple structure to promote clarity and reduce misinterpretation.

All telephone questionnaire interviews were conducted by a sole research coordinator to limit variation in questioning and were performed across a four-week span from June to July 2023 to reach as many eligible patients as possible. The research investigator attempted to call each patient three times to administer the questionnaire and varied the time of the telephone call at least once in the afternoon or evenings. Patients were free to complete or decline to take the questionnaire.

Data collection

Data and contact information were collected through review of the patients’ medical charts, which included demographic details, medical history, consultation notes, diagnosis information, prescribed treatments, and clinical outcomes. This resource also provided us with hospital identification numbers, age, and race.

The mainstay of our data collection strategy was a scripted telephone questionnaire. Through the questionnaire, we were able to gather information on the number of children participants had, their household income, education level, and marital status. In addition, participants were read five statements querying the motivation behind electing to undergo vasectomy surgery (see Appendices). Participants were asked to rate each statement on a scale of 1-5 (1 - strongly disagree, 2 - somewhat disagree, 3 - neither agree nor disagree, 4 - somewhat agree, 5 - strongly agree). 

Data analysis

Data analysis was conducted using SAS 9.4 statistical software (SAS Institute Inc., Cary, North Carolina, United States). Fundamental descriptive statistics, including mean and standard deviation (SD) for continuous variables, and proportions and frequencies for categorical variables, were calculated. We further explored the relationship between the reported motivations for choosing a vasectomy and demographic characteristics. The following statistical tests were used for analysis: chi-square for categorical data to identify any significant associations, and Spearman’s rank correlation analysis was employed to examine the Likert-scale data.

## Results

We attempted to survey 164 subjects; 69 (42%) men did not answer the survey phone calls, and five (3%) patients could not be contacted due to an invalid or disconnected phone number on file. We were able to contact 90 men (55%), with 30 men declining to participate. Of the possible 90 participants, 60 men were successfully surveyed, yielding a response rate of 36.6%. Participants and non-participants did not have significant differences in mean age (36.9 ± 7.8 vs. 34.2 ± 7.9 years, p=0.119). Participant demographics are reported in Table 1.

**Table 1:** Participant demographics (N=60) GED, general education diploma

Characteristics	Frequency (Percentage)
Race	
White	60 (100)
Highest level of education attained	
High School/GED	22 (36.7)
Associate's degree	7 (11.7)
Bachelor's degree	17 (28.3)
Graduate degree	14 (23.3)
Income	
< $25,000	1 (1.7)
$25,001-$55,000	11 (18.3)
$55,001-$85,000	19 (31.7)
$85,001-$115,000	8 (13.3)
>$115,000	18 (30.0)
Declined to answer	3 (5.0)
Marital status	
Single	9 (15.0)
Dating and cohabiting	4 (6.7)
Married	40 (66.7)
Separated/Divorced/Widowed	7 (11.7)
Number of children	
0	12 (20.0)
1	10 (16.7)
2 or 3	34 (56.7)
4 or 5	4 (6.7)

Several factors were identified related to the Dobbs decision that had a significant impact on the participants’ decision to undergo a vasectomy. A quarter (25%) of the participants that completed the survey somewhat agreed or strongly agreed that access to abortion care (15/60), or the Supreme Court ruling to overturn *Roe v. Wade* (15/60) influenced their decision to undergo vasectomy, and one third (33%, n=19) of the men surveyed somewhat agreed or strongly agreed that access to maternal healthcare influenced their decision (Table 2).

**Table 2:** Survey responses (N=60) *n=59; one respondent’s answer was “Not Applicable”

Survey Statement	Strongly Disagree, n (%)	Somewhat Disagree, n (%)	Neither Agree or Disagree, n (%)	Somewhat Agree, n (%)	Strongly Agree, n (%)
Access to maternal healthcare influenced my decision to undergo vasectomy	29 (48.3)	4 (6.7)	8 (13.3)	6 (10.0)	13 (21.7)
Previous pregnancy complications influenced my decision to undergo vasectomy*	41 (68.3)	1 (1.7)	8 (13.3)	2 (3.3)	7 (11.7)
Access to abortion care influenced my decision to undergo vasectomy	41 (68.3)	0 (0)	4 (6.7)	5 (8.3)	10 (16.7)
The Supreme Court overturning *Roe v. Wade* influenced my decision to undergo vasectomy	39 (65.0)	2 (3.3)	4 (6.7)	3 (5.0)	12 (20.0)
My religious beliefs influenced my decision to undergo vasectomy	55 (91.7)	1 (1.7)	4 (6.7)	0 (0)	0 (0)

**Figure 1:**
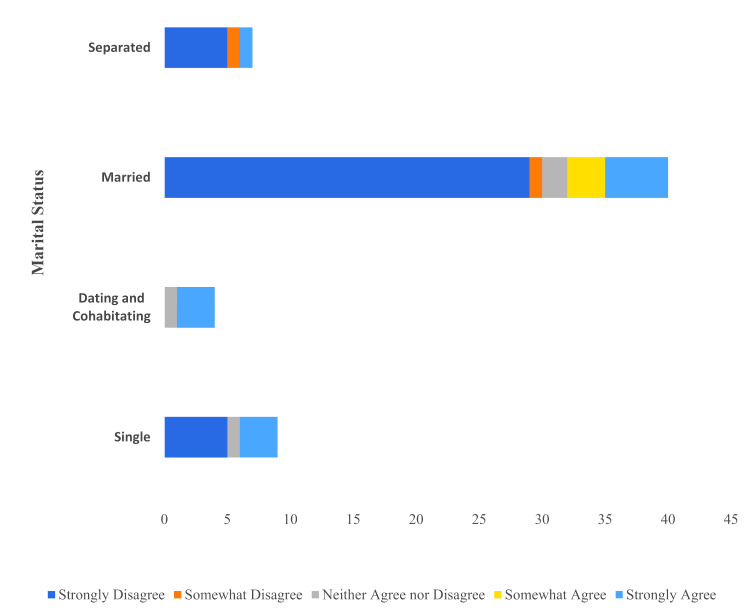
Distribution according to relationship status of participants whose decision to undergo vasectomy was influenced by the overturning of Roe v. Wade A total of 60 survey responses to "The Supreme Court overturning Roe v. Wade influenced my decision to undergo vasectomy" by relationship status: Separated category (n=7), married category (n=40), dating and cohabitating (n=4), and single (n=9). Separated category contains formerly married (i.e., separated, divorced, or widowed).

There was a significant negative correlation between marital status and whether the *Dobbs* decision impacted participants’ decision making for a vasectomy (Spearman’s rank correlation, r (59) = (-0.25), p=0.05). Three of the four participants (75%) who were dating and cohabiting strongly agreed that the change to *Roe v. Wade* had an impact on their decision to undergo a vasectomy. Married participants (30/40, 75%) were less likely to report that the Dobbs decision impacted their decision-making (Figure 1).

**Figure 2:**
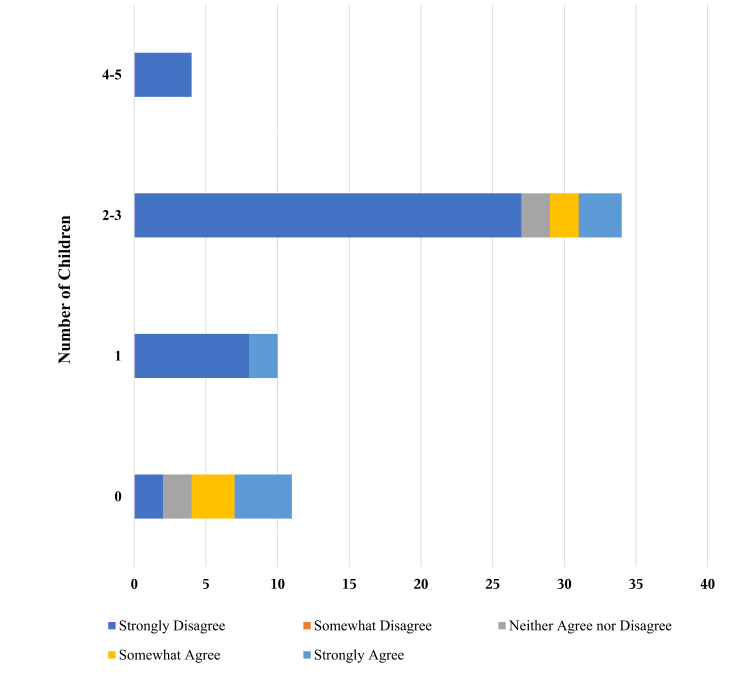
Relationship between the number of children and access to abortion care as an influence for the decision to undergo vasectomy. A total of 60 survey responses to "Access to abortion care influenced my decision to undergo vasectomy" were distributed according to the number of children categories: 0 (n=12), 1 (n=10), 2-3 (n=34), and 4-5 (n=34).

The number of children a participant had was significantly related to access to abortion care as a factor attributing to the participants’ decision to undergo a vasectomy. This was indicated by a significant negative correlation between the number of children and access to abortion care as an important factor in the vasectomy decision-making process (Spearman’s rank correlation, r(59) = (-0.24), p=0.05). Seven of the 38 participants with no children or one child (18%) reported access to abortion care as a strong influencer of the decision to undergo vasectomy, while 10 of the 22 participants with two or three children or four or five children (45%) did not report that this was a factor influencing their decision (Figure 2).

**Figure 3:**
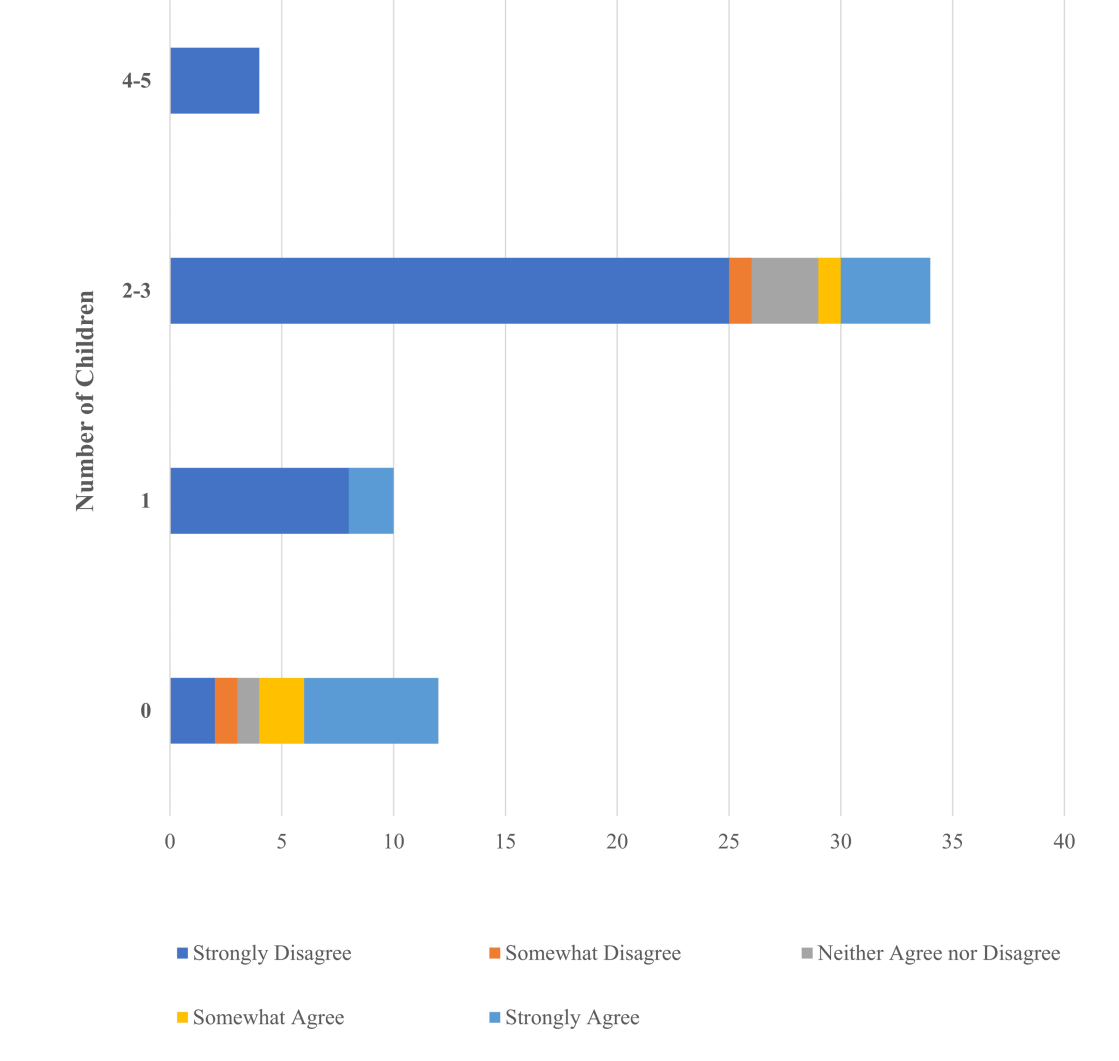
Relationship between the number of children and the overturning of Roe v. Wade as an influence on the decision to undergo vasectomy. Survey responses (n=60) to "The Supreme Court overturning Roe v. Wade influenced my decision to undergo vasectomy" were distributed by number of children categories: 0 (n=12), 1 (n=10), 2-3 (n=34), and 4-5 (n=4).

The number of children a participant had was also associated with the change to *Roe v. Wade* as an important factor in deciding to undergo a vasectomy. Participants with no children or one child (10/22, 45%) and participants with two or three children (5/34, 14.7%) reported concerns regarding the Supreme Court decision to overturn *Roe v. Wade* as motivating factors (Figure 3).

## Discussion

With this study, we aimed to better understand the factors that influence a man’s decision to undergo a vasectomy in the post-*Dobbs *era of reproductive healthcare. We identified several factors related to the changing reproductive healthcare landscape that were associated with impacted decision-making in participants who had undergone vasectomy. Participants who were cohabiting were more likely to identify the Supreme Court’s decision to overturn *Roe v. Wade* as a factor that influenced their decision to undergo a vasectomy. Participants with no children or one child reported that access to maternal health care and access to abortion care were associated factors that weighed into their reproductive healthcare decision-making. A clearer understanding of motivating factors for vasectomy may help urologists better counsel men seeking vasectomy to combat misinformation and support the intended permanence of the procedure.

On June 24, 2022, the United States Supreme Court’s ruling in *Dobbs v. Jackson Women’s Health Organization* overturned the precedent-setting *Roe v. Wade *decision, leading to an avalanche of trigger laws that limited access to abortion care. As of the summer of 2023, 13 states had banned abortion completely, and additional states had legislation or legislation pending that is hostile to abortion [12]. The post-*Dobbs* healthcare landscape affects the field of urology uniquely, as urologists are intimately involved in male reproductive healthcare [13]. Public interest in vasectomy has recently increased. Almost immediately after *Roe v. Wade* was overturned, Google searches for “vasectomy” skyrocketed with the highest search rate for the term in the past five years [14,15]. The number of vasectomies performed annually by urology practices has increased compared with previous years [16]. Furthermore, online discussions about vasectomy may sometimes frame the procedure as more reversible than intended. This can contribute to misconceptions among some men who view vasectomy as a temporary contraceptive option rather than a permanent form of sterilization [17]. Misrepresentation of vasectomy reversal is often shared with many posts characterizing it as an “easy procedure.” This erroneous conclusion may be contributing to some men’s desire to undergo a vasectomy, although this factor was not investigated in this study. Of note, longer median wait time from consultation to vasectomy has been reported in the post-*Dobbs* era (56 v 52 days) [11]. This may indicate increased wait time for surgery, or rather, heightened individual contemplation prior to pursuing definitive sterilization. 

Several studies have found that more young men are seeking vasectomies in the post-*Dobb* era. There has been a reported 20-22.4% increase in vasectomy procedures performed in several post-*Dobbs* investigations [7-10]. Additionally, it has been shown that the median age of men receiving a vasectomy has been lower in a post-*Dobbs* cohort (38 years vs. 39 years) [11]. A separate analysis showed a similar trend with median age post *Dobbs* at 35 years median age versus 38 years. It has been reported in a cohort of 142 individuals that 16.9% of men post *Dobbs* were childless compared to 8.6% pre *Dobbs* [16]. These studies also indicate an increase in child-free men receiving vasectomies.

Due to this reported upswing of childless men seeking vasectomy, we divided the participants with 0-1 children noted in our survey into separate groups (no children vs. one child). This allowed a better understanding of patient motivations, as we were able to identify several factors associated with the *Dobbs* decision that separated men with no children from those with at least one child, pertaining to their decision to undergo a vasectomy.

In participants with no children, the Supreme Court’s decision overturning *Roe v. Wade* was associated with the decision to undergo a vasectomy. Furthermore, access to abortion healthcare was associated with the childless participants who underwent a vasectomy and participated in our survey. Among adults under 30 years of age, 66% support legal abortion in all or most cases [18]. Perhaps support of and access to legal abortion influences younger men’s decision to undergo a vasectomy, accounting for the increased number of younger childless men undergoing the procedure. The influence of participant age at the time of vasectomy was not specifically investigated in this study, but remains an area of future interest. Questionnaire responses from participants with one child revealed that access to abortion care was associated with decision-making, but paradoxically, the overturning of *Roe v. Wade* was not. This discrepancy may be due to the participants’ perception of abortion. “Termination of a pregnancy for medical reasons,” such as genetic abnormality, appears to be perceived differently than “abortion.” Termination for medical reasons appears more publicly accepted, as this scenario is not perceived as abortion of an unwanted child [19,20].

Future interests include further exploration of the decision-making to undergo elective sterilization in men with no children, as this population has had an increase in vasectomies. Investigating the impact of age on the decision to undergo a vasectomy would also be beneficial for patients and providers to understand. Further avenues of research comprise vasectomy reversal rates via vasovasostomy or vasoepididymostomy in the post-*Dobbs *era.

Limitations include social-desirability bias regarding a sensitive topic that included questions about policy as well as reproductive healthcare; non-response bias distorting observations due to participants available during this time period; and the fact that this was a single-institution study conducted in a state (West Virginia) that has a history of banning abortion dating back to 1849 [21], which may limit generalizability. To reduce social-desirability and non-response biases, the questionnaire was administered by a male research investigator, repeating to participants that the questionnaire would be kept in total confidentiality, and the questionnaire was designed using concise, neutrally worded, closed-ended Likert-scale items to minimize respondent burden and encourage participation, particularly given the sensitive nature of reproductive decision-making. Although our facility is a large referral center with a catchment area of four adjacent states with similar demographics and beliefs, the single-institute design limits the generalization of our results to a wider population. Biases also remain possible, as patients who completed the survey may have differed from those who did not participate in ways not measurable from data extracted from the medical record or have been subject to recall bias. Accordingly, findings should be interpreted as reflective and exploratory of surveyed participants rather than the entire eligible cohort of vasectomy patients.

## Conclusions

Our questionnaire identified several characteristics associated with participants who reported greater consideration of vasectomy in the post-*Dobb *era. Factors such as the number of children, access to maternal healthcare, access to abortion care, and the Supreme Court’s decision to overturn *Roe v. Wade* were reported as influential, particularly in participants without children. These insights provide insight into factors that may contribute to vasectomy decision-making. As we better understand the motivations underlying participants’ decisions to undergo vasectomy, we may improve counseling regarding the intended permanence of the procedure and better contextualize changes in the reproductive healthcare landscape.

## Figures and Tables

**Figure 4:**
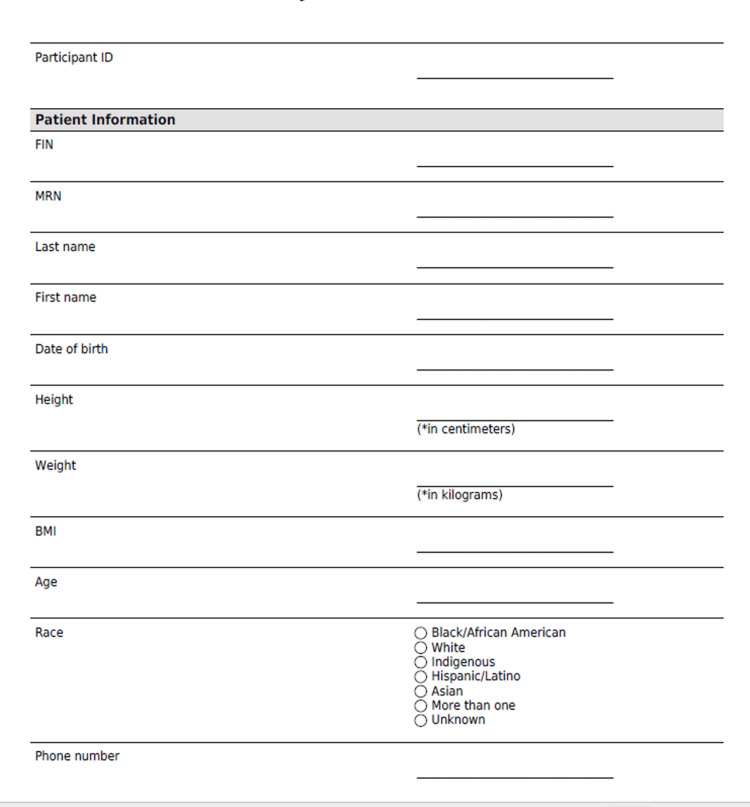
Page 1 of the questionnaire

**Figure 5:**
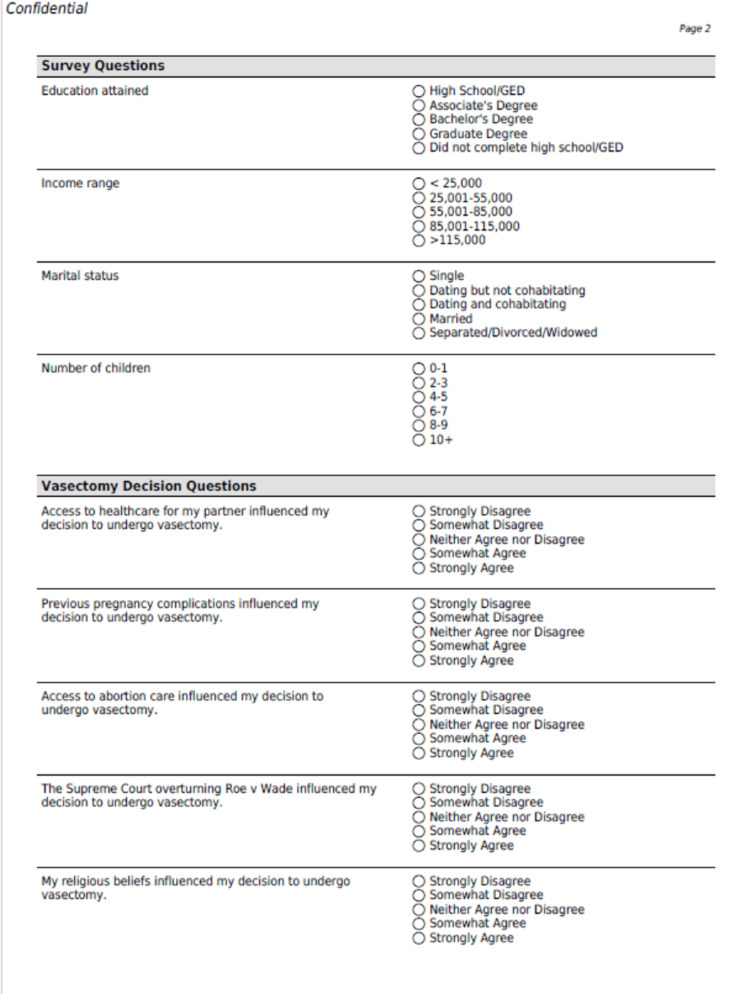
Page 2 of the questionnaire

**Figure 6:**
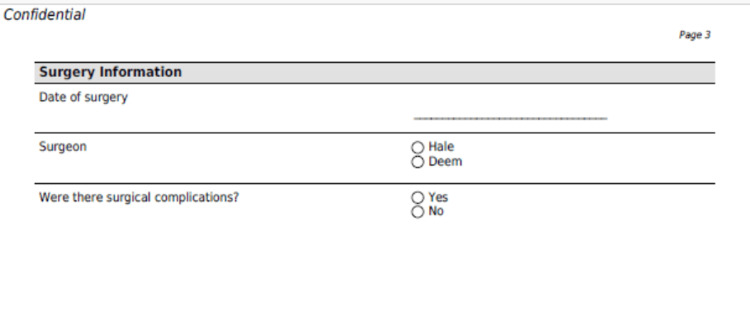
Page 3 of the questionnaire

## Appendices

Study questionnaire
